# Standardization of *teff* (*Eragrostis teff*) injera making process conditions for better physicochemical and sensory quality

**DOI:** 10.1002/fsn3.4006

**Published:** 2024-02-07

**Authors:** Gizachew M. Bikila, Yetenayet B. Tola, Chala G. Kuyu

**Affiliations:** ^1^ Department of Post‐harvest Management Jimma University College of Agriculture and Veterinary Medicine Jimma Ethiopia

**Keywords:** baking condition, injera, optimization, pre‐baking process

## Abstract

Injera is a type of flatbread that is fermented, naturally leavened, and native to Ethiopia. However, injera quality can vary depending on the processing steps used, even if the same variety of teff is used. This research was conducted to optimize the prebaking processing and baking conditions to produce better quality teff injera suitable for industrial and export purposes. Four experiments were conducted to optimize the injera‐making process. The first two phases focused on optimizing the prebaking processing conditions (fermentation temperature and time, absit mixing ratio, absit cooking time, and secondary fermentation time). The best physicochemical qualities were obtained at a primary fermentation temperature of 25°C for 64 h, an 8% mixing ratio of absit with 10 min of cooking, and a secondary fermentation time of 4 h. In the third phase, baking temperature (195 ± 5, 215 ± 5, 235 ± 5, and 255 ± 5°C) and time (1, 2, and 3 min) were evaluated. The results showed that the best response variables were obtained at a temperature of 255 ± 5°C for 2 min or 235 ± 5°C for 3 min. Finally, the optimized conditions were validated on five different varieties [DZ‐Cr‐387, DZ‐Cr‐2124, white (T‐BT), white (T‐GK), and sergegna teff (T‐E)] of teff grain. The results indicated that the optimized conditions could produce better quality and consistent teff injera on a large commercial scale, which would suit both local and export markets.

## Graphical abstract

The traditional‐based processing approach influences the quality of Injera. To standardize the traditional‐based processing approach. Four consecutive experiments were conducted, and optimized conditions were obtained. The processing conditions were further validated to fit for industrial and export purposes. The result is an input to standardize Injera making to industry‐level procedure.

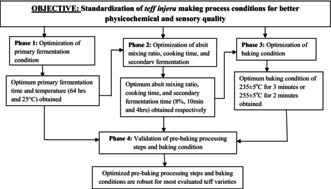

## 1 INTRODUCTION

*Teff* (*Eragrostis teff*) is a cereal crop widely cultivated in various agroecology regions of Ethiopia (Jemal et al., 2018). It covers over one million hectares of land yearly, or about 28.4% of the total area cultivated for cereal crops. Recently, teff grain has gained popularity worldwide as an exceptionally nutritious and healthy grain (Minten et al., 2016). Teff grain is receiving global attention due to its nutritional importance and gluten‐free content, making it an increasingly important dietary component for individuals who suffer from gluten intolerance (Boka et al., 2013). The dietary fiber content of teff is several times higher (4.5%) than that of maize, sorghum, wheat, and rice (Hager et al., 2012). It is also rich in micronutrients such as calcium, copper, iron, manganese, zinc, thiamin, vitamin K, digestible proteins, and essential amino acids. Compared to other cereals, teff is low in sodium, saturated fat, and cholesterol (Boka et al., 2013).

In Ethiopia, teff is traditionally used to make various baked products, with a significant portion of teff production used to make Injera (Awol et al., 2023; Kuyu et al., 2024; Minten et al., 2013). Injera is a common and widely consumed staple food in Ethiopia and abroad. It is typically made in most Ethiopian kitchens using traditional processing steps inherited from the parents. Due to their specific experiences, different people use different prebaking procedures and baking conditions to make Injera. However, variations in prebaking processing steps and baking conditions can result in variations in physicochemical and sensory quality, even if the same variety of teff is used (Yetneberk et al., 2005). Furthermore, the preparation of Injera takes several days due to the lack of well‐standardized prebaking and baking conditions that can fit a wide range of teff varieties (Mulaw & Tesfaye, 2017).

Various attempts have been made to investigate the potential of teff for developing value‐added food products, as well as teff processing and injera shelf life extension aspects (Boka et al., 2013; Girma et al., 2013; Teshome et al., 2017; Yoseph, 2019). However, optimum prebaking and baking process conditions are scarce to produce consistent and better quality teff injera.

Optimum primary fermentation time and temperature, optimized absit‐making process conditions (absit mixing ratio, cooking time, and secondary fermentation time), and baking conditions (temperature and time) for better injera quality have yet to be reported. These gaps need to be scientifically studied and optimized to produce consistent‐quality injera to support large‐scale commercial production and export of the product to other countries. With existing traditional processing and the absence of well‐written and documented standard processing methods, teff injera's wide use and consumption are restrained in international markets. Therefore, developing the optimum processing method will enhance the broad commercialization and use of teff injera worldwide. This, in turn, creates better market opportunities for teff grower farmers and those associated with its value chain.

## 2 MATERIALS AND METHODS

### 2.1 Sample collection and preparation

The experiments were conducted in four consecutive phases, with Kuncho (DZ‐Cr‐387) and red (DZ‐Cr‐2124) teff varieties collected from the Bishoftu Agricultural Research Center of the Ethiopian Institute of Agricultural Research (EIAR) for the first three phases. For the last phase of experimentation, three additional teff varieties (white (T‐BT), white (T‐GK), and sergegna (T‐E)) were randomly purchased from the local market. The spontaneous culture for back slopping for all phases of the study was collected from different houses and stored to be used at different stages of the experiments.

The teff grain samples were manually cleaned by sifting and winnowing to remove stones, dust, light materials, and other extraneous materials. The cleaned teff grains were then milled using a small‐scale commercial hammer mill to produce fine flour. After milling, the flour was sifted through a 0.5‐mm sieve (Abebe et al., 2015) and stored in polyethylene plastic bags at room temperature until further use.

### 2.2 Experimental design and treatment combination

**FIGURE 1:**
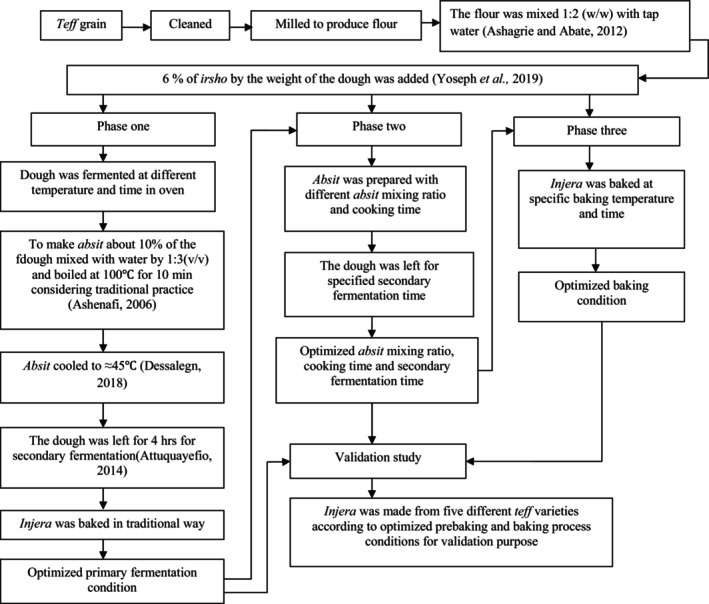
*Injera*‐making process flow diagram for all phases of the study.

Overall, the experimental setup for the four consecutive phases of the study is depicted in Figure 1. To conduct the study, the researchers used a response surface methodology called the central composite design for the first two phases. They adjusted the experimental ranges for both phases based on preliminary work and previous literature data from Ashagrie & Abate (2012) and Attuquayefio (2014). In the first phase, the researchers varied the primary fermentation temperature and time to make dough, with a range of 25–38°C and 24–96 h, respectively. This resulted in 14 experimental runs. For the second phase, the researchers considered the absit mixing ratio (5%–15%), cooking time (5–15 min), and secondary fermentation time (2–6 h) to optimize the secondary fermentation condition. This phase generated 20 experimental runs.

In the third phase of the study, the researchers evaluated the baking temperature and time using a temperature‐regulated baking plate. The experimental design was a completely randomized block design (RCBD) with 4 × 3 factorial arrangements in three replications. The factors included baking temperature (195 ± 5, 215 ± 5, 235 ± 5, and 255 ± 5°C) and time (1, 2, and 3 min).

In the final phase, a validation study was conducted to verify the robustness of the optimized prebaking and baking process conditions. The experimental design was a completely randomized block design (RCBD) with 5 × 2 factorial arrangements in three replications. The factors included teff varieties with five levels (DZ‐Cr‐387 (A), DZ‐Cr‐2124 (B), white (T‐BT) (C), white (T‐GK) (D), and sergegna teff (T‐E) (E)), and optimum baking conditions with two levels (255 ± 5°C for 2 min and 235 ± 5°C for 3 min).

### 2.3 Data collected

#### 2.3.1 Percent of carbon dioxide formed during primary fermentation

Carbon dioxide gas formed during primary fermentation was measured using a CO_2_ meter (Oxybaby M + i O_2_/CO_2_, E7, Germany) at the end of fermentation.

#### 2.3.2 Determination of eye numbers per unit area of injera

The number of eyes on the surface of Injera was determined by counting the number of eyes on a portion of the sample measuring 3 × 3 cm and dividing the total number of eyes counted from four different portions of Injera, each having a surface area of 9 cm^2^ (3 × 3 cm) Yasin (2021).

#### 2.3.3 Determination of rollability

The procedure used by Yasin (2021) was followed to determine rollability. A 12 cm long and 2 cm wide sample was cut from a full Injera and wrapped or rolled around a 2 cm diameter wooden dowel. The rollability score was rated on a scale of 1–5, with one indicating that the Injera breaks immediately after one roll and cannot be rolled, two indicating that it breaks in two rolls, three indicating that it breaks in three rolls, four indicating that it breaks in four rolls, and five indicating that it does not crack and is very flexible.

#### 2.3.4 Determination of extensibility

A texture analyzer (TA, U.K.) was used to determine the extensibility of Injera. Before the evaluation, the analyzer was adjusted with a 1 mm/s test speed, 1.5 mm/s pre‐test speed, 10 mm/s post speed, 60 mm bottom distance, and target distance bottom. The length of the Injera sample was 10 cm, measured by holding and pulling the two ends of the Injera on the machine.

#### 2.3.5 Determination of pH and moisture

The pH and moisture content of the experimental samples were determined using the AOAC (2000) method 925.09.

#### 2.3.6 Sensory quality

The sensory quality of the samples was evaluated by 50 untrained panelists using a five‐point hedonic scale ranging from 1 to 5, where 5 represents “Like extremely,” 4 represents “Like moderately,” 3 represents “Neither like nor dislike,” 2 represents “Dislike moderately,” and 1 represents “Dislike extremely.”

### 2.4 Statistical analysis

For the first and second phases of the study, the data were analyzed and modeled using Design Expert® version 6.0.2, Minneapolis, USA, to generate second‐degree polynomial models with response surface effects. Contour plots were generated to visualize the combined effects of two factors on the response while keeping the third factor at its median value. For the third and fourth phases of the study, the results were analyzed using analysis of variance (ANOVA) with the Minitab statistical computer software program version 19. Differences were determined by the Tukey's test when *p*‐values were significant at a 5% probability level.

## 3 RESULTS AND DISCUSSION

### 3.1 Phase one: Optimization of primary fermentation time and temperature of teff dough

The study analyzed the effects of primary fermentation time and temperature on selected physicochemical quality parameters. The results indicated significant differences (*p* < .05) for all quality parameters analyzed, as shown in Table 1.

**TABLE 1:** Regression coefficients of predicted quadratic polynomial and percentage of precision (*R* ^2^) for some physical and chemical quality parameters of teff *injera*.

Source	CO_2_ (%)	No of E/cm^2^	Roll/2 cm	Ext. (N)	pH	MC (%)
Model	93.81**	1.53**	−1.52**	0.66**	5.12**	59.10**
Block	0.39	0.21	−0.09	0.00	−0.01	0.09
A	0.41*	0.41**	0.10**	0.00*	−0.03**	−0.12
B	−0.20*	0.00	0.17	0.05*	−0.03	0.19*
A^2^	−0.01**	0.00**	0.00**	0.00**	0.00**	0.00**
B^2^	−0.01	0.00	0.00	0.00*	0.00	0.00
AB	0.01	0.00	0.00	0.00	0.00*	0.00
Lack of fit	0.00	0.50	0.108	0.10	0.11	0.61
*R*‐Sq	89.32%	89.32%	95.36%	87.82%	98.67%	82.55%
*R*‐Sq(adj)	80.82%	80.17%	91.39%	77.38%	97.54%	67.60%

*Note*: **Significant at 1%, *Significant at 5%.

Abbreviations: A, fermentation Time (h); B, Fermentation Temperature (°C); Ext. (N), Extensibility (N); MC, moisture content (%); No of E/cm^2^, Number of eyes/cm^2^; Roll/2 cm, Rollability/2 cm.

#### 3.1.1 Percent of carbon dioxide formation

The CO_2_ concentration of the dough was analyzed concerning primary fermentation time and temperature. The results showed that the highest CO_2_ concentration (96.7%) was observed in dough fermented at 22°C for 60 h, while the lowest concentration was observed in a sample fermented at 32°C for 9 h (Table 2). The study found that lower CO_2_ production was observed at a relatively higher temperature and extended fermentation time, possibly due to a less favorable temperature and the conversion of produced CO_2_ to different compounds with extended fermentation time. This observation is consistent with previous studies by Michel et al. (2016) and Yetneberk et al. (2004).

**TABLE 2:** Mean value of some physicochemical quality parameters of *teff injera*.

RO	Block	A	B	CO_2_ (%)	No of E/cm^2^	Roll/2 cm	Ext. (N)	pH	MC (%)
1	1	60	32	93.00	16.04	4.40	1.48	3.38	59.80
2	1	96	38	81.00	15.16	4.12	1.46	3.39	63.56
3	1	24	25	94.50	8.91	2.85	1.43	3.85	61.75
4	1	96	25	83.00	15.73	4.30	1.36	3.31	62.59
5	1	60	32	94.00	16.50	4.55	1.48	3.43	62.60
6	1	24	38	91.50	13.60	3.45	1.30	3.67	64.00
7	1	60	32	94.20	14.85	4.65	1.47	3.37	62.82
8	2	60	32	93.90	16.04	4.68	1.48	3.42	61.75
9	2	60	22	96.70	16.15	4.80	1.42	3.45	58.90
10	2	9	32	78.00	4.73	2.65	1.29	4.12	65.35
11	2	111	32	79.30	14.73	4.05	1.39	3.37	63.25
12	2	60	32	94.00	16.44	4.70	1.47	3.40	63.80
13	2	60	41	88.50	16.23	4.25	1.39	3.39	63.00
14	2	60	32	95.25	16.53	4.45	1.48	3.38	61.00

Abbreviations: A, Fermentation Time (h); B, Fermentation Temperature; Ext. (N), Extensibility (N); MC, Moisture content (%); No of E, Number of eyes/cm^2^; RO, run order; Roll, Rollability (2 cm of the wood roller diameter).

**FIGURE 2:**
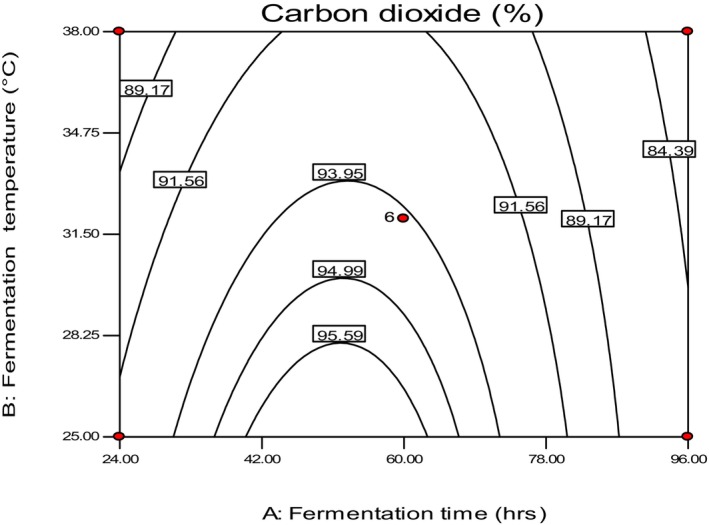
The fermentation time and temperature affect carbon dioxide formed during primary fermentation conditions.

The study also found that the highest CO_2_ production occurred between 50 and 55 h of fermentation, with an optimum temperature level between 25 and 28°C (Figure 2). This trend in CO_2_ production could be attributed to the activity of microflora responsible for fermentation, whether in an inactive or dormant stage, which might be associated with these optimum conditions. This finding is consistent with a previous study by Heitmann et al. (2018). The study highlights the importance of primary fermentation time and temperature in regulating CO_2_ production during dough fermentation.

#### 3.1.2 Number of eyes

Table 2 shows that the total number of eyes per unit area ranged from 4.43 to 16.53/cm^2^. The highest values (16.04–16.23 eyes/cm^2^) were recorded from samples fermented at 32°C for 60 h, while the sample fermented at 32°C for 9 h had the lowest number of eyes (4.43/cm^2^). This suggests that fermentation time is essential in determining the number of eyes formed per unit area. The study found a correlation between the number of eyes and CO_2_ produced during fermentation, which is consistent with the work of Pyle (2005), who stated that CO_2_ resulting from the fermentation stage plays a significant role in pore development or a porous structure.

**FIGURE 3:**
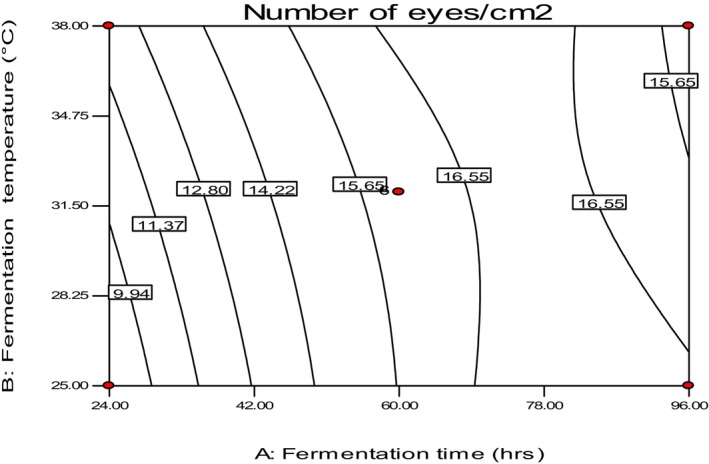
The effect of fermentation time and temperature on the number of eyes.

The study also found that the number of eyes increased with increasing fermentation time, regardless of temperature, and decreased for extended fermentation time, approximately 85 h (Figure 3). This suggests that the accumulation of gas during fermentation time, rather than temperature, is the primary factor in determining the number of eyes, which corresponds to better quality injera. Other researchers have also highlighted that carbon dioxide significantly affects the formation of pores on the surface of the injera. The greater the amount of CO_2_ or gas bubbles in the fermented batter, the greater the number of pores formed on the injera (Mengesha et al., 2022; Mihrete, 2019).

#### 3.1.3 Rollability of injera

Table 2 shows the roller wood's rollability values, which ranged from 2.65 to 4.80/2 cm in diameter. The highest value for rollability was observed in the sample fermented at 22°C for 60 h, while the sample fermented at 32°C for 9 h had the lowest rollability values. The study found that short fermentation times and higher fermentation temperatures can damage rollability.

**FIGURE 4:**
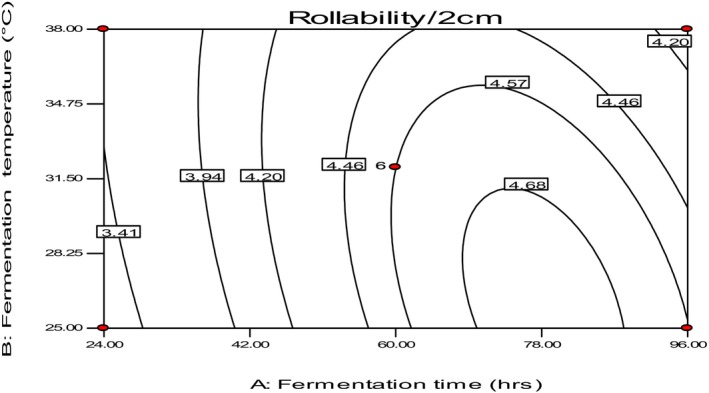
The effect of fermentation time and temperature on rollability of *injera*.

The optimum condition for rollability was found to be at a temperature range of 25–29°C with a fermentation time of 70–80 h (Figure 4). As fermentation time extended, the components of teff flour could be degraded by microorganisms, which can contribute to elasticity. The lower value for a shorter fermentation time might be due to less chance of modification of the starch by the microflora, which can result in cracking or tearing of the Injera during rolling, as observed in a previous study by Yetneberk et al. (2004).

#### 3.1.4 Extensibility of injera

The extensibility value of Injera ranged from 1.29 to 1.48 N, with the highest value measured in the sample fermented at 32°C for 60 h and the lowest value in the sample fermented at 32°C for 9 h (Table 2). Similar to rollability, the extensibility values showed similar trends and were highly influenced by fermentation time rather than temperature. The dough should ferment for a sufficient time to degrade the structural components of teff flour, such as proteins, starch, and hemicelluloses, and produce higher exopolysaccharides that are responsible for the elasticity of Injera (Attuquayefio, 2014). Ruhmkorf et al. (2012) also stated that exopolysaccharides produced during fermentation could act as hydrocolloids and contribute to the elastic texture of Injera.

**FIGURE 5:**
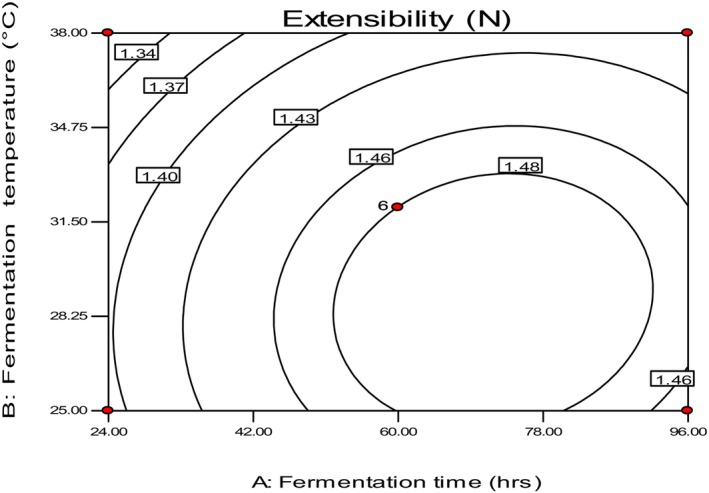
The effect of fermentation time and temperature on extensibility.

The study found that the extensibility of Injera attained a higher value for fermentation time between 60 and 80 h and fermentation temperature of 25 and 32°C (Figure 5). Lazaridou et al. (2007) stated that the protein matrix is a major determinant of the important rheological characteristics of dough during primary fermentation, such as elasticity, extensibility, resistance to stretch, and gas‐holding ability, which might be critically attained at these ranges. This protein matrix could be affected at a higher temperature and decrease the matrix contribution to the elasticity of the Injera.

#### 3.1.5 The pH of injera

The study found that the pH of fresh injera samples ranged from 3.31 to 4.12 (Table 2), consistent with what has been reported in other literature for teff injera (Ashagrie & Abate, 2012; Attuquayefio, 2014). The highest pH value (4.12) was measured in the sample fermented at a temperature of 32°C for 9 h, while the sample fermented at 25°C for 96 h had the lowest pH value (3.31) (Table 2). From a food safety perspective, a lower pH is always better as it inhibits the growth of bacteria, although it might alter the taste and flavor of Injera.

**FIGURE 6:**
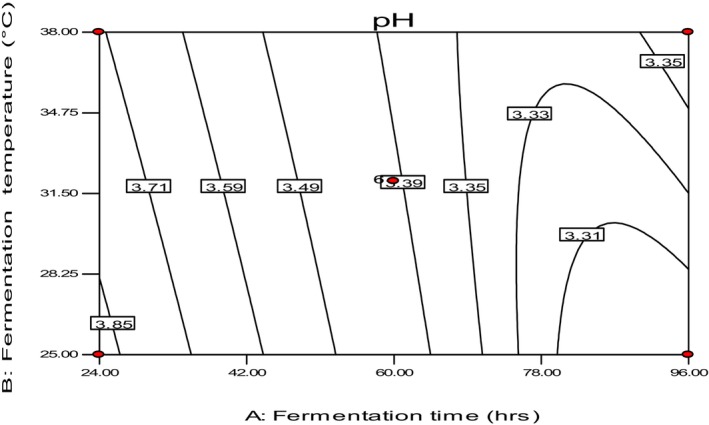
The effect of fermentation time and temperature on pH.

The study observed that as primary fermentation time and temperature increased, the pH values decreased due to the action of lactic acid bacteria (LAB) on the teff batter (Figure 6). The pH value depended on the lactic acid content in the fermented batter on the day of baking, indicating that as fermentation time and temperature increased, the pH value of Injera decreased (Attuquayefio, 2014; Urga & Narasimha, 1997). The teff's moisture, amylose, and starch contents significantly affect pH values depending on the primary fermentation time and temperature (Sahlin, 1999). Overall, the study highlights the importance of primary fermentation time and temperature in regulating the pH of Injera.

#### 3.1.6 The moisture content of injera

The study measured the moisture content of Injera for each run of the experiment, which ranged between 58.90 and 65.35% (Table 2). The measured values align with the moisture content specification (58%–63%) set by the standard Ethiopian agency (ESA, 2013), except for treatments conducted at 32°C for 9 and 24 h. The values are also consistent with other literature results on teff injera, such as the 65.23% reported by Attuquayefio (2014). The highest (65.23%) value of the moisture content of fresh Injera was obtained at 32°C for 9 h compared to other treatments, while the sample fermented at 22°C for 60 h had the lowest (58.90%) moisture content value (Table 2).

**FIGURE 7:**
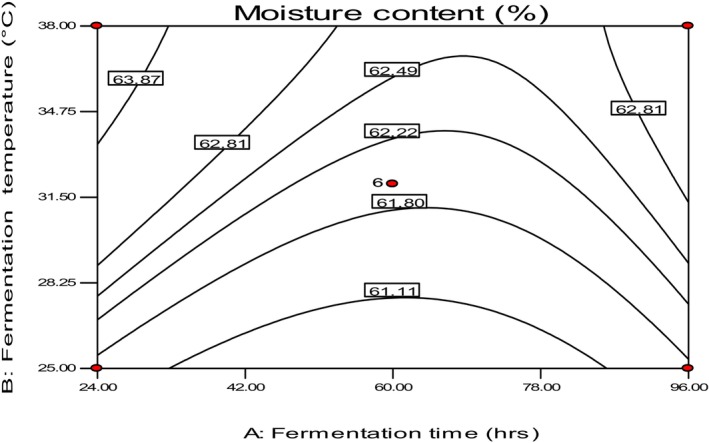
The effect of fermentation time and temperature on moisture content.

As shown in Figure 7, the study found that fermentation temperature mainly influenced the moisture content compared to fermentation time. The possible reason for the increment in moisture content with fermentation temperature might be starch gelatinization at high fermentation temperature—the higher starch gelatinization occurred due to higher fermentation temperature due to batters' higher water‐holding capacity. Pinnavaia and Pizzirani (1998) also reported a good correlation between the water‐holding capacity and gelatinization degree of certain starchy foods. Overall, the study highlights the importance of fermentation temperature in regulating the moisture content of Injera.

#### 3.1.7 Optimum primary fermentation condition

The study conducted numerical optimization by incorporating appropriate constraints to optimize the independent and dependent variables. The optimized values were 64 h of primary fermentation time and 25°C of primary fermentation temperature, resulting in a desirable value of 0.86. The optimized physicochemical quality parameters at these optimum conditions were 95.56% for carbon dioxide concentration, 16.06/cm^2^ for the number of eyes, 4.60/2 cm for rollability, 9.30% for extensibility, 1.45 N for pH, 3.40 for texture, and 60.44% for moisture content. These optimized values can be used as a reference for producing high‐quality Injera with desirable physicochemical properties.

### 3.2 Phase two: Optimization of absit mixing ratio, cooking time, and secondary fermentation time

In this phase, the optimized primary fermentation time and temperature from phase one (64 h and 25°C) were used as input to optimize the intended objective. The ANOVA results showed significant differences (*p* < .05) for all parameters conducted in this phase, as shown in Table 3. This indicates that the optimized independent variables significantly impacted the physicochemical properties of the Injera. The results of this phase can be used to further improve the quality of Injera by fine‐tuning the fermentation conditions.

**TABLE 3:** Regression coefficients of predicted quadratic polynomial and percentage of precision (*R* ^2^) for some physicochemical quality parameters of *teff injera.*

Source	*N* of E/cm^2^	Roll/2 cm	Ext. (N)	pH	MC (%)
Model	−1.31**	1.27**	0.91**	3.85**	72.49**
Block	0.41	0.17	0.01	0.01	−0.04
A	2.88**	0.39**	0.06**	−0.02**	−0.72**
B	0.32	0.16*	0.03	−0.03*	−0.51
C	2.20	0.65	0.08	−0.03	−0.76
A^2^	−0.15**	−0.02**	0.00**	0.00	0.00
B^2^	−0.02	−0.01	0.00**	0.00	0.01
C^2^	−0.27	−0.07*	−0.01	0.00	0.01
AB	−0.01	−0.01	0.00	0.00*	0.02
AC	−0.03	0.00	0.00	0.00	0.04
BC	0.04	0.00	0.00	0.00	0.02
Lack of fit	0.00	0.051	0.066	0.068	0.32
*R*‐Sq	86.48%	88.04%	90.22%	88.36%	93.02%
*R*‐Sq(adj)	71.45%	74.75%	79.36%	75.43%	85.26%

*Note*: **Significant at 1%, *Significant at 5%.

Abbreviations: A, Mixing ratio of absit (%); B, Cooking time of absit (min); C, Secondary fermentation time (h); Ext. (N), extensibility (N); MC, Moisture content (%); Roll, Rollability/2 cm wooden roller of diameter.

#### 3.2.1 The number of eyes

The mean values of eye numbers ranged from 6.23 to 16.6/cm^2^, as shown in Table 4. These values were slightly higher than the findings of Desalegn (2018), who reported the number of eyes between 11.32 and 13.65 eyes/cm^2^ for teff injera. The highest value of 16.6 eyes/cm^2^ was obtained from Injera made with a 10% absit mixing ratio, 10 min of cooking time, and 4 h of secondary fermentation time. On the other hand, the lowest value of 6.23 eyes/cm^2^ was obtained from the sample baked at an 18% absit mixing ratio, cooked for 10 min, and 4 h of secondary fermentation time. Similarly, a lower absit mixed ratio value of 2% with a 10‐min cooking time and a 4‐h secondary fermentation time resulted in similar eye numbers of 6.5 eyes/cm^2^.

**TABLE 4:** Mean values of some physicochemical quality parameters for absit mixing making process of *teff injera*.

RO	Blocks	A	B	C	No of E/cm^2^	Roll/2 cm	Ext. (N)	pH	MC (%)
1	Block 1	4	10	10	16.55	4.72	1.53	3.40	61.55
2	Block 1	4	10	10	16.58	4.55	1.48	3.43	61.85
3	Block 1	6	5	5	15.66	4.38	1.52	3.49	63.00
4	Block 1	4	10	10	16.59	4.73	1.48	3.40	61.00
5	Block 1	2	15	5	10.60	4.15	1.36	3.50	63.25
6	Block 1	6	15	15	7.50	2.89	1.32	3.41	63.56
7	Block 1	6	5	15	14.55	4.45	1.40	3.48	63.87
8	Block 1	2	5	15	13.43	3.96	1.28	3.45	63.95
9	Block 1	2	15	15	8.56	2.96	1.24	3.47	62.50
10	Block 1	4	10	10	16.54	4.75	1.51	3.43	58.85
11	Block 1	2	5	5	11.48	3.25	1.41	3.54	64.00
12	Block 1	6	15	5	10.35	3.95	1.45	3.41	62.30
13	Block 2	4	10	10	16.60	4.74	1.50	3.40	59.90
14	Block 2	4	18	10	6.23	2.25	1.38	3.52	65.23
15	Block 2	4	10	18	14.87	4.25	1.24	3.42	63.00
16	Block 2	4	10	2	14.31	4.73	1.38	3.46	62.45
17	Block 2	1	10	10	9.87	4.25	1.35	3.41	60.52
18	Block 2	7	10	10	15.54	4.25	1.52	3.43	64.95
19	Block 2	4	2	10	6.50	2.95	1.22	3.48	62.45
20	Block 2	4	10	10	16.60	4.75	1.57	3.41	61.00

Abbreviations: A, absit mixing ratio (%); B, absit cooking time (min); C, Secondary fermentation time (h); Ext. (N), extensibility (N); MC, Moisture content (%); RO, Run order; Roll, Rollability/2 cm wooden roller of diameter.

**FIGURE 8:**
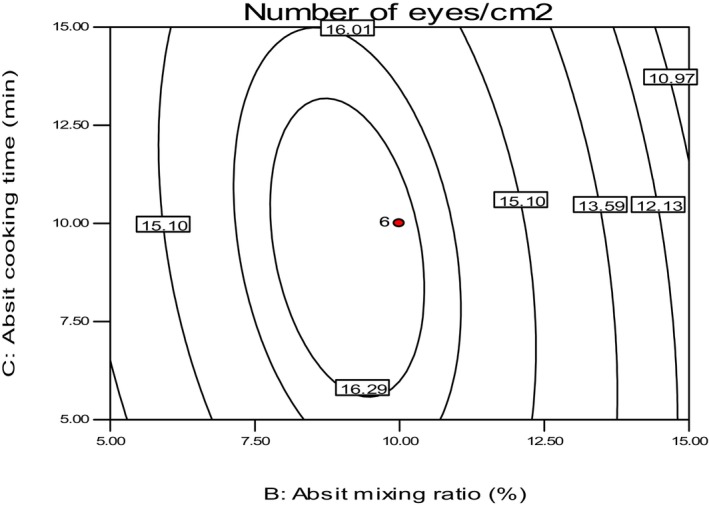
The effect of *absit* mixing ratio and cooking time on a number of eyes of *injera*.

Cooking absit breaks down starch components to form more sugars for yeast activity, leading to more fermentation and CO_2_ production, as shown in Figure 8. A higher absit mixing ratio produced small‐sized eyes that were difficult to count. This observation is consistent with the findings of Attuquayefio (2014), who stated that Injera with large, unevenly spaced eyes or those with tiny eyes are considered poor quality.

The study identified the optimum range for absit preparation to achieve a high number of eyes per unit area after 4 h of secondary fermentation to be between 8 and 10% absit mixing ratio with a cooking time of 5–11 min. It was observed that an increase in the mixing ratio above 10% resulted in a decrease in the number of eyes formed per unit area. This finding is consistent with the report of Ashenafi (2006), who stated that Injera baked with less absit or an overdose amount of absit ratio has fewer eyes. Therefore, it is essential to maintain the optimum absit mixing ratio and cooking time to achieve the desired number of eyes in Injera.

#### 3.2.2 Rollability

The mean values of rollability for different treatments ranged from 2.25 to 4.75/2 cm, as shown in Table 4. The sample baked with 10% absit mixing ratio, 10 min of cooking time, and 4 h of secondary fermentation time at ambient temperature had the highest mean value (4.75/2 cm) for rollability. On the other hand, the samples baked with a high percentage of absit ratios (18%), cooked for 10 min, and fermented for 4 h of secondary fermentation had the lowest rollability value. These differences were likely due to the variation in the absit mixing ratio, which made it break easily during the rolling time.

**FIGURE 9:**
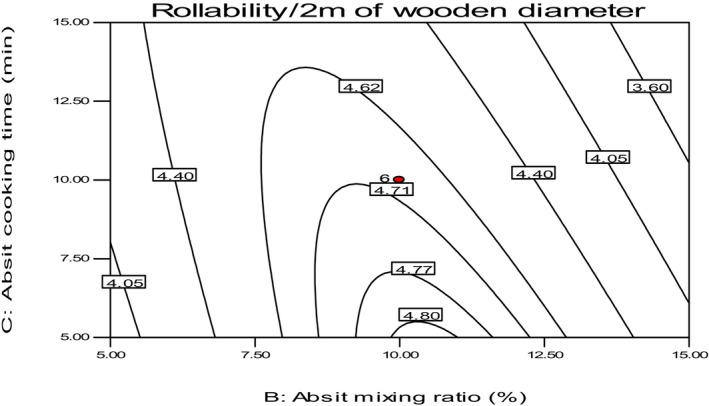
The effect of *absit* mixing ratio and cooking time on rollability of *injera*.

The study found that the rollability of Injera decreased with increasing absit mixing ratio and cooking time, as shown in Figure 9. The results indicated that both the lowest (2%) and the highest (18%) levels of absit ratio caused a significant change in the rollability of Injera, with the optimum being close to a 10% absit mixing ratio. This finding is consistent with the report of Houben et al. (2012), who stated that the proper amount of gelatinized starch added to the original fermented dough increases the viscosity of the batter and is responsible for the elasticity of baked Injera. Ashenafi (2006) also indicated that Injera baked without absit or with less absit is easily breakable.

#### 3.2.3 Extensibility

The study found that the sample baked with 10% absit mixing ratio, cooked for 10 min, and had 4 h of secondary fermentation time had the highest value (1.57 N) for extensibility, as shown in Table 4. On the other hand, the samples baked with the highest (2%) absit mixing ratio, cooked for 10 min, and 4 h of secondary fermentation time had the lowest extensibility value. Attuquayefio (2014) reported that the elastic modulus of injera samples increased gradually with the increasing viscosity of batters by adding some amounts of absit to the original dough, and this condition could be used to increase the elasticity/extensibility of baked Injera. This finding is consistent with similar reports in the literature.

**FIGURE 10:**
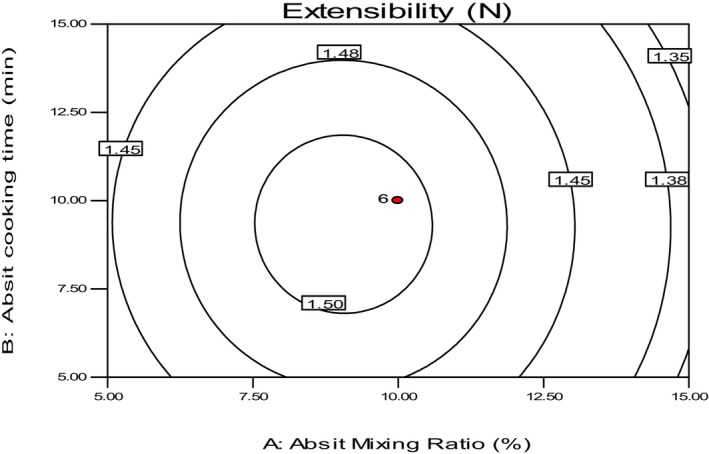
The effect of *absit* mixing ratio and cooking time on the extensibility of *injera*.

The viscosity, texture, and rheological properties of many cooked starchy foods are affected by the interaction of starch with protein or changes in the structure due to bacterial or enzymatic activities or other processes (Bultosa & Taylor, 2004; Wang et al., 2015). The result of this study shows that extensibility moderately decreased with an increased mixing ratio of the absit from the fermented dough, as indicated in Figure 10. Therefore, it is essential to maintain the optimum absit mixing ratio and cooking time to achieve the desired extensibility of Injera.

#### 3.2.4 pH of injera

**FIGURE 11:**
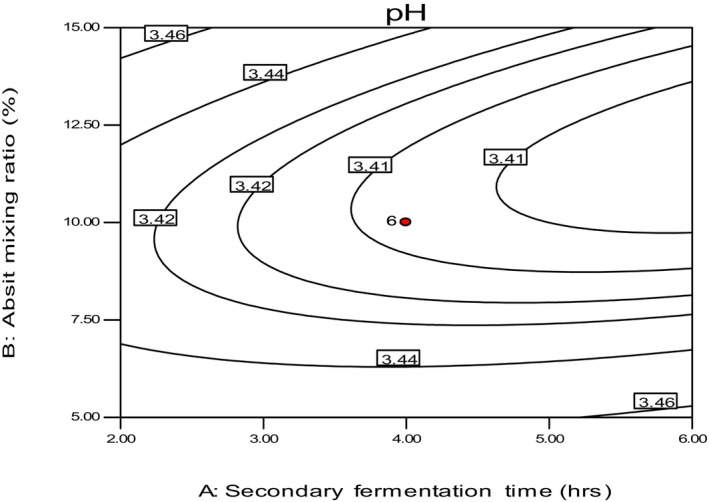
The effect of *absit* mixing ratio and secondary fermentation time on pH.

The pH of the injera samples in this study ranged between 3.4 and 3.54, as shown in Table 4. These values are consistent with the result of Attuquayefio (2014), who recorded a pH of 3.73 on teff injera. The sample baked with a 5% absit mixing ratio from the fermented dough, 5 min of cooking time, and 2 h of secondary fermentation time had the highest pH value (3.54). The sample baked with a 15% absit mixing ratio, 10 min of cooking time, and 4 h of secondary fermentation time had the lowest pH value (3.4). The variation in pH was likely due to the higher fermentation time and absit mixing ratio, which caused the dough to become more acidic and decreased pH after baking. The pH readings of Injera in different kinds of literature are variable due to the variation in fermentation time (Urga & Narasimha, 1997; Yigzaw et al., 2004). Figure 11 indicates that the pH values decreased with the increase in the absit mixing ratio.

#### 3.2.5 Moisture content

**FIGURE 12:**
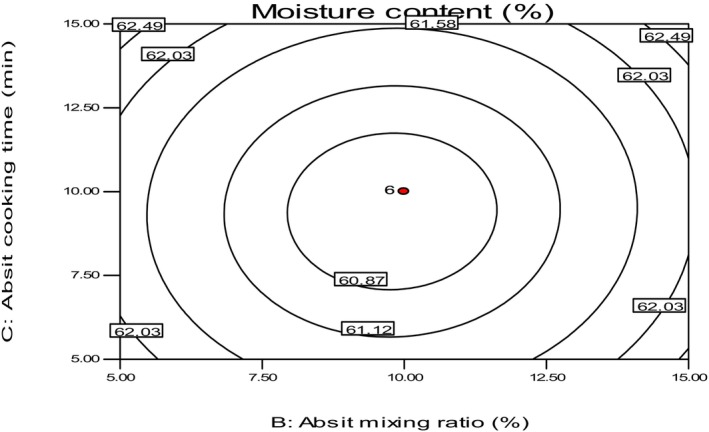
The effect of *absit* mixing ratio and cooking time on the moisture content of *injera*.

In this study, the moisture content of fresh injera samples (as soon as they were baked) ranged from 58.850% to 65.23%, as shown in Table 4. This result is consistent with the study by Desalegn (2018), which reported a moisture content range of 61.04%–62.32% for teff injera. The sample with an absit mixing ratio of 18%, cooked for 10 min, and left for 4 h of secondary fermentation time had the highest moisture content (65.23%). In comparison, the sample with a 10% absit mixing ratio, 10 min of cooking time, and 4 h of secondary fermentation time had the lowest moisture content value. This variation was likely due to the low and high absit added to the original dough. The higher the water content in the batter due to the high absit added, the higher the moisture content of the final product. Starch gelatinization is strongly affected by the water content of food products (Pyle, 2005). As indicated in Figure 12, Injera's moisture content increased with the absit mixing ratio increase compared to secondary fermentation time.

#### 3.2.6 Optimized absit mixing ratio, cooking time, and secondary fermentation time

The study conducted numerical optimization by setting constraints for each response. As a result, the optimum values for independent variables were 8% of absit mixing ratio, 10 min of cooking time, and 4 h of fermentation time, with an overall desirability value of 0.89. The optimum values for physicochemical quality parameters at these conditions were also determined to be 16.46/cm^2^ for eye number, 4.68/2 cm for rollability, 1.5 N for extensibility, 3.42 for pH, and 60.82% for moisture content. These values represent the ideal conditions for producing Injera with the desired physicochemical quality parameters.

### 3.3 Phase three: Evaluation of baking time and temperature

In phase three of the study, the optimized primary fermentation time and temperature from phase one (64 h and 25°C) and the optimized absit mixing ratio (8%), absit cooking time (10 min), and secondary fermentation time (4 h) from phase two were used as inputs to optimize the intended objective. The analysis showed that the interaction effect of baking temperature and time significantly influenced (*p* < .05) all physicochemical quality parameters, as shown in Table 5. This indicates that the baking temperature and time are important factors to consider in achieving Injera's desired physicochemical quality parameters. Therefore, it is essential to control the baking temperature and time carefully to achieve the desired quality of Injera.

**TABLE 5:** Mean values of selected physicochemical quality parameters of *injera*‐based temperature and time.

A (min)	B (°C)	No of E/cm^2^	Ext. (N)	pH	MC (%)
1	195 ± 5	10.50 ± 0.19^g^	1.26 ± 0.04^fg^	3.42 ± 0.04^g^	66.11 ± 0.65^a^
	215 ± 5	11.30 ± 0.15^fg^	1.30 ± 0.11^f^	3.43 ± 0.04^g^	65.60 ± 0.62^ab^
	235 ± 5	13.10 ± 0.82^cde^	1.36 ± 0.14^ef^	3.50 ± 0.08^ef^	64.90 ± 0.53^abc^
	255 ± 5	13.40 ± 0.30^def^	1.39 ± 0.09^cd^	3.54 ± 0.06^de^	63.30 ± 0.47^bcd^
2	195 ± 5	12.30 ± 0.74^ef^	1.40 ± 0.06^bc^	3.46 ± 0.07^fg^	65.30 ± 0.54^ab^
	215 ± 5	14.80 ± 0.12^cde^	1.45 ± 0.07^bcd^	3.50 ± 0.04^ef^	64.80 ± 0.67^abc^
	235 ± 5	15.80 ± 0.17^abc^	1.52 ± 0.08^ab^	3.55 ± 0.06^d^	64.30 ± 0.51^abcd^
	255 ± 5	16.80 ± 0.43^a^	1.55 ± 0.10^a^	3.73 ± 0.06^ab^	60.50 ± 0.52^de^
3	195 ± 5	16.30 ± 0.11^ab^	1.45 ± 0.04^bcd^	3.53 ± 0.06^de^	64.20 ± 0.42^abc^
	215 ± 5	15.50 ± 0.48^bcd^	1.53 ± 0.06^ab^	3.64 ± 0.06^bc^	61.70 ± 0.58^cd^
	235 ± 5	16.90 ± 0.47^a^	1.55 ± 0.08^a^	3.74 ± 0.06^a^	60.50 ± 0.41^de^
	255 ± 5	15.80 ± 0.38^abc^	1.52 ± 0.08^ab^	3.75 ± 0.06^a^	59.00 ± 0.43^e^
	CV	6.54	4.64	2.55	3.73

*Note*: Means with different superscripts (a, b, c) in the column are significantly different at *p* < .05 assessed by Tukey test.

Abbreviations: A, Baking time (min); B, Baking temperature (°C); Ext. (N), Extensibility (N); MC, Moisture content (%); No of E, Number of eyes/cm^2^.

#### 3.3.1 Number of eyes

The study found that Injera baked at 195 ± 5°C for 3 min, 235 ± 5°C for 2 and 3 min, and 255 ± 5°C for 2 and 3 min had the highest number of eyes (15.80–16.9/cm^2^), while the lowest eye number (10.50–11.30/cm^2^) was observed in Injera baked at 195 ± 5°C and 215 ± 5°C for 1 min (Table 5). These results are consistent with the work of Cherinet (1993), who determined an appropriate number of eyes on the surface of Injera to be 11–15 eyes/cm^2^. During the baking of Injera, gas bubbles are used to create nuclei, which result in the formation of eyes on the surface of the Injera. The creation of nuclei requires sufficient baking temperature and time. Lower baking temperatures and shorter baking times result in fewer gas bubbles and, consequently, fewer eyes on the surface of the Injera. This finding is consistent with the work of Pyle (2005), who stated that the dissolved CO_2_ released from the batter during the baking of Injera could contribute to pore development in Injera.

#### 3.3.2 Extensibility of injera

The study found that the highest mean value of extensibility (1.5–1.55 N) was recorded from Injera baked at a temperature of 215 ± 5°C for 3 min and 235 ± 5 and 255 ± 5°C for 2 and 3 min (Table 5). The lowest mean values (1.26–1.36 N) were recorded from Injera baked at 195 ± 5, 215 ± 5 and 235 ± 5°C for 1 min. Similar to eye numbers, higher baking temperatures for 2–3 min were associated with higher extensibility than lower temperatures and shorter baking times. The variation in extensibility could be due to insufficient starch gelatinization resulting from lower baking temperatures and shorter baking times. This finding is consistent with the work of Parker et al. (1989), who stated that during the baking of Injera, the starch in the batter gelatinizes to form a steam‐leavened and porous starch matrix, which contributes to the elastic property of Injera.

#### 3.3.3 The pH of injera

The study found that the highest pH of fresh Injera (3.73–3.75) was measured from Injera baked at 255 ± 5°C for 2 min and 235 ± 5 and 255 ± 5°C for 3 min (Table 5). The lowest pH (3.42–3.46) was recorded at 195 ± 5 and 215 ± 5°C for 1 min and 195 ± 5°C for 2 min. In this study, there was an inverse relationship between pH and moisture content; the moisture content decreased as pH increased. This finding is consistent with the work of Attuquayefio (2014), who reported an inverse relationship between pH and moisture content for commercially available Injera. The Ethiopian Standard Agency (ESA, 2013) has set the pH of teff injera to be in the range of 3.45–4.0, and the pH values obtained in this study were not significantly different from the set values of ESA, except for Injera baked at 195 ± 5 and 215 ± 5°C for 1 min (Table 5).

#### 3.3.4 The moisture content of injera

The study found that the highest mean value of moisture content (64.20%–66.11%) of fresh injera samples was obtained from Injera baked at 195 ± 5, 215 ± 5 and 235 ± 5°C for 1 min, 195 ± 5, 215 ± 5 and 235 ± 5°C for 2 min and 195 ± 5°C for 3 min (Table 5). However, the lowest mean value (59%–60.5%) was measured from the sample baked at 255 ± 5°C for 2 min and 235 ± 5 and 255 ± 5°C for 3 min. As baking temperature and time increase, the moisture content of Injera decreases (Table 5). Moisture content changes can affect Injera's physicochemical quality, individually or collectively. This finding is consistent with the work of Assefa (2018), who stated that the moisture content of baked Injera depends on the amount of moisture during baking, baking temperature, and time.

### 3.4 Phase four: Validation of prebaking processing steps and baking conditions

The study used five different teff varieties to produce Injera and validated the optimized prebaking and baking process conditions. The results showed that there were no significant differences (*p* > .05) among four teff varieties in terms of various physicochemical properties, except for Injera baked from red teff (Table 6). Injera made from red teff (DZ‐Cr‐2124) had few eye numbers, less extensibility, and low moisture content compared to Injera from other teff varieties. This variation could be due to the inherent properties of the red teff variety, which differ in color and chemical composition of flour from the other varieties. Gebru et al. (2020) conducted a study on the nutritional composition and health benefits of teff. The study indicated that the processing conditions for teff grains vary depending on the teff variety, which aligns with the current findings, especially the variations observed in the red grain variety.

**TABLE 6:** Mean values of selected physicochemical quality parameters for validation study.

*Teff* varieties	Optimum baking conditions	No of E/cm^2^	Ext. (N)	pH	MC (%)
DZ‐Cr‐387	255 ± 5°C for 2 min	16.59 ± 0.15^a^	1.55 ± 0.04^a^	3.47 ± 0.04^a^	65.19 ± 0.17^a^
	235 ± 5°C for 3 min	16.07 ± 0.20^a^	1.51 ± 0.04^a^	3.46 ± 0.04^a^	64.98 ± 0.08^a^
DZ‐Cr‐2124	255 ± 5°C for 2 min	13.88 ± 0.17^b^	1.44 ± 0.04^b^	3.45 ± 0.04^a^	64.23 ± 0.31^ab^
	235 ± 5°C for 3 min	13.13 ± 0.24^c^	1.44 ± 0.04^b^	3.43 ± 0.13^a^	63.39 ± 0.23^b^
White teff T‐BT	255 ± 5°C for 2 min	16.50 ± 0.12^a^	1.54 ± 0.07^a^	3.47 ± 0.06^a^	65.02 ± 0.21^a^
	235 ± 5°C for 3 min	16.25 ± 0.27^a^	1.52 ± 0.08^a^	3.44 ± 0.08^a^	64.78 ± 0.40^a^
White teff T‐GK	255 ± 5°C for 2 min	16.43 ± 0.14^a^	1.53 ± 0.08^a^	3.43 ± 0.10^a^	65.01 ± 0.24^a^
	235 ± 5°C for 3 min	16.12 ± 0.28^a^	1.53 ± 0.08^a^	3.44 ± 0.08^a^	64.69 ± 0.46^a^
Sergna teff	255 ± 5°C for 2 min	16.32 ± 0.17^a^	1.52 ± 0.08^a^	3.44 ± 0.06^a^	64.90 ± 0.24^a^
	235 ± 5°C for 3 min	16.22 ± 0.29^a^	1.53 ± 0.06^a^	3.44 ± 0.08^a^	64.32 ± 0.36^a^
CV		7.52	2.64	0.73	0.92

*Note*: DZ‐Cr‐387 = Quncho teff, DZ‐Cr‐2124 = red teff, optimum baking conditions (255 ± 5°C for 2 min and 235 ± 5°C for 3 min). Means with different superscripts (a, b, c) in the column are significantly different at *p* < .05 assessed by Tukey test.

Abbreviations: Ext. (N), Extensibility (N); MC, Moisture content (%); No of E/cm^2^, Number of eyes/cm^2^.

The study found that there were no significant differences (*p* > .05) in terms of sensory properties for Injera prepared from four teff varieties and optimized conditions (objectives one to three), except for Injera made from the red teff variety. Injera made from red teff had lower scores for color, taste, texture, eye distribution (eye uniformity), and overall acceptability compared to other treatments (Table 7). However, the evaluated sensory qualities of Injera were numerically close to each other. This suggests that the optimized prebaking processing steps and baking conditions developed are robust for these four varieties, but further optimization is needed for red teff.

**TABLE 7:** Mean values of sensory quality analysis as affected by *teff* varieties and baking temperature and time.

*Teff* varieties	Optimum baking conditions	Color	Taste	Texture	Eye distribution	Overall acceptability
DZ‐Cr‐387	255 ± 5°C for 2 min	4.41 ± 0.06^a^	4.01 ± 0.06^a^	4.40 ± 0.00^a^	4.46 ± 0.09^a^	4.76 ± 0.17^a^
	235 ± 5°C for 3 min	4.35 ± 0.09^a^	4.2 ± 0.09^a^	4.34 ± 0.12^a^	4.30 ± 0.16^a^	4.70 ± 0.05^a^
DZ‐Cr‐2124	255 ± 5°C for 2 min	3.43 ± 0.18^b^	4.0 ± 0.18^b^	3.20 ± 0.18^b^	3.71 ± 0.17^b^	3.23 ± 0.13^b^
	235 ± 5°C for 3 min	3.11 ± 0.30^b^	4.01 ± 0.32^b^	3.23 ± 0.24^b^	3.55 ± 0.12^b^	3.24 ± 0.14^b^
White teff T‐BT	255 ± 5°C for 2 min	4.43 ± 0.12^a^	3.6 ± 0.12^ab^	4.35 ± 0.12^a^	4.40 ± 0.23^a^	4.67 ± 0.14^a^
	235 ± 5°C for 3 min	4.18 ± 0.09^a^	4.0 ± 0.09^a^	4.21 ± 0.24^a^	4.31 ± 0.17^a^	4.64 ± 0.35^a^
White teff T‐GK	255 ± 5°C for 2 min	4.19 ± 0.14^a^	3.8 ± 0.14^ab^	4.38 ± 0.21^a^	4.23 ± 0.26^a^	4.70 ± 0.30^a^
	235 ± 5°C for 3 min	4.28 ± 0.09^a^	3.9 ± 0.09^a^	4.36 ± 0.20^a^	4.31 ± 0.17^a^	4.69 ± 0.14^a^
Sergna teff	255 ± 5°C for 2 min	4.36 ± 0.14^a^	3.9 ± 0.14^a^	4.20 ± 0.31^a^	4.44 ± 0.17^a^	4.65 ± 0.24^a^
	235 ± 5°C for 3 min	4.16 ± 0.22^a^	3.9 ± 0.22^a^	4.31 ± 0.16^a^	4.17 ± 0.21^a^	4.71 ± 0.18^a^
CV		11.06	8.88	11.63	7.62	3.50

*Note*: DZ‐Cr‐387 = Quncho teff, DZ‐Cr‐2124 = red teff and optimum baking conditions (255 ± 5 for 2 min and 235 ± 5 for 3 min). Means with different superscripts (a, b, c) in the column are significantly different at *p* < .05 assessed by Tukey test.

## 4 CONCLUSION

The traditional processing methods used in most Ethiopian households to produce teff injera can negatively impact the product's physicochemical and sensory quality. This study found that Injera baked using different prebaking and baking conditions showed different responses regarding physicochemical and sensory quality. The best quality injera was produced using a primary fermentation temperature of 25°C for 64 h, and the fermented dough was mixed with absit at a mixing ratio of 8%, cooked for 10 min, and held for a secondary fermentation time of 4 h at ambient temperature. The baking temperature was the most significant factor in determining the quality of Injera, with a temperature of 235 ± 5°C for 3 min or 255 ± 5°C for 2 min producing the best results. Overall, primary fermentation time, absit mixing ratio, and baking temperature were the most significant factors in determining the quality of Injera compared to other studied factors.

The optimized conditions for teff injera production were further validated using different teff flours, and the results showed no significant differences in the evaluated quality parameters. This suggests that processing conditions, rather than teff varieties, mainly influence the consistency and quality of teff injera. Therefore, optimizing the process conditions for teff injera production can effectively enhance its utilization in Ethiopia and worldwide. The optimized prebaking processing steps and baking conditions developed in this study could transform the traditional injera‐making process into a more standardized industry‐level procedure for large‐scale production. This could improve teff injera's quality and consistency and increase its availability to consumers.

## AUTHOR CONTRIBUTIONS

**Gizachew M. Bikila:** Conceptualization (supporting); data curation (equal); formal analysis (equal); investigation (lead); methodology (equal); writing – original draft (lead); writing – review and editing (equal). **Yetenayet B. Tola:** Conceptualization (lead); data curation (equal); formal analysis (equal); funding acquisition (equal); investigation (equal); methodology (equal); resources (equal); software (equal); supervision (lead); validation (equal); visualization (equal); writing – original draft (equal); writing – review and editing (equal). **Chala G. Kuyu:** Conceptualization (equal); data curation (equal); formal analysis (lead); funding acquisition (equal); investigation (equal); methodology (equal); project administration (equal); resources (equal); software (equal); supervision (equal); validation (equal); writing – original draft (equal); writing – review and editing (lead).

## CONFLICT OF INTEREST STATEMENT

All authors declare no conflict of interest.

## Data Availability

Data are available upon request from the corresponding author.
